# 

**Published:** 1913-11

**Authors:** 


PUBLISHED MONTHLY. SUBSCRIPTION $1.00

### ATLANTA JOURNAL-RECORD OF MEDICINE

LIBRARY
DEC-10 1913
SURGEON GENERAL'S OFFICE

Successor to { Atlanta Medical and Surgical Journal, Established 1855. and Southern Medical Record, Established 1870. } 60th Year

Vol. LX. ATLANTA, GA., NOVEMBER, 1913. No. 8

